# STN‐DBS in the 1950s? Carl Wilhelm Sem‐Jacobsen's Missing Films Recovered

**DOI:** 10.1002/mds.29217

**Published:** 2022-09-02

**Authors:** Espen Dietrichs

**Affiliations:** ^1^ Department of Neurology, Oslo University Hospital and Institute of Clinical Medicine University of Oslo Oslo Norway

## Author Role

E.D. did all the background research and wrote the manuscript.

## Full financial disclosures for the previous 12 months

E.D. has received honoraria for lectures from AbbVie and NordicInfucare during the past 12 months.

**FIG 1:**
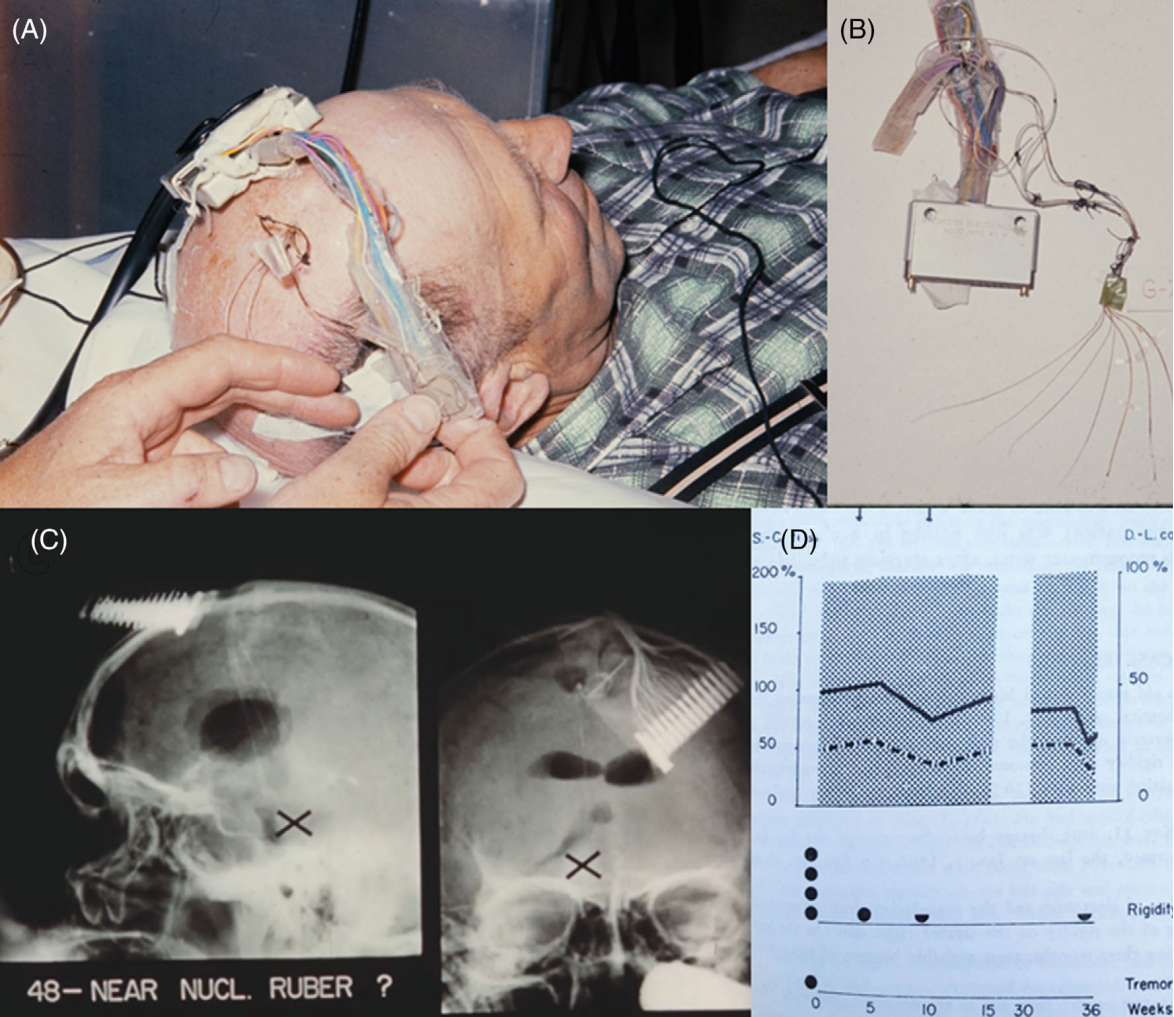
(**A**) Each patient had several electrodes for DBS and depth recordings implanted for several weeks. The picture shows a patient during this test period, with one cable from each intracranial electrode. (**B**) Sem‐Jacobsen's self‐constructed electrodes. Four to eight leads, each with several contacts for stimulation and registration, were implanted into one or both hemispheres. (**C**) X‐rays from one of the patients in the film, showing electrode positions. Sem‐Jacobsen assumed that the stimulation site in this patient was close to the red nucleus. (**D**) Diagram from Hartviksen and Sem‐Jacobsen^5^ showing clinical improvement in one patient during 6 weeks of DBS (from weeks 4 to 10, start and stop of stimulation period indicated by small arrows above the diagram). Solid and dashed lines show the time used for two standard motor tasks and indicate that the patient's motor speed (bradykinesia) improves during DBS. The slightly increasing height of the dotted area indicates a minor improvement in quality of life. Larger dots in the lower part of the diagram indicate that both rigidity (severe, indicated by four dots before treatment) and tremor (moderate, one dot) are considerably reduced during DBS. The right part of the panel shows the same tests after chemical lesion, but the site of the lesion has not been specified.

In 1956, the Norwegian neurophysiologist Carl Wilhelm Sem‐Jacobsen (1912–1991) was appointed as head of his own neurophysiology unit at Gaustad Psychiatric Hospital in Oslo, Norway (now part of Oslo University Hospital). Originally he was hired to do depth electrode stimulations and recordings to improve precision for lobotomies in psychiatric patients, but he soon changed focus to test patients with Parkinson's disease (PD) before thalamotomy or similar lesion surgery.^1^ Through the late 1950s and 1960s a series of Parkinson patients received deep brain stimulation (DBS) at Gaustad as a method for target identification before chemical lesioning.^2^, ^3^ According to Hariz et al.^4^ these were the first attempts ever with DBS in PD. Neurosurgeons from Rikshospitalet (The National Hospital) performed implantations of special electrodes designed by Sem‐Jacobsen (Fig. 1A–C). Each patient received up to at least eight electrodes, each with multiple contacts for stimulation and recordings. The patients had these electrodes for 4–6 weeks before chemical lesioning.^3^ Based on the observations from stimulation and recordings during this period the team at Gaustad chose what they considered to be the optimal target and then injected a toxin (ethyl cellulose in ethanol) to make the chemical lesion. Sem‐Jacobsen's electrodes had a separate channel for injections, so chemical lesions were made without a second operation.^3^

Even though the aim was to improve lesion surgery and DBS was performed only for a few weeks in each patient, a good therapeutic response was found during the stimulation period.^5^ Mortality and morbidity were low both during stimulation and after chemical lesioning. One of 30 operated patients died, 3 weeks after the chemical lesion and possibly from unrelated causes.^6^

Carl Wilhelm Sem‐Jacobsen was probably more an inventor of medical technical devices than a medical researcher. His methods and some of his observations were described in a 1966 article,^2^ and in more detail in his exhaustive monography.^3^ However, few of his results were presented in peer‐review medical journals. Another problem is that much of his material was destroyed after Sem‐Jacobsen was accused of doing mind‐control experiments for the CIA and his family was haunted by investigating journalists. He was later officially absolved by a Norwegian Government Hearing Committee, but only long after his death.^1^

Sem‐Jacobsen presented his results on film in the Scandinavian Neurology Congress in 1962. But his valuable film documentation has also been missing for almost 60 years. With help from Sem‐Jacobsen's family I recently found the missing films and a huge collection of forgotten photos in a barn in rural Norway.^1^ The films and photos document the operations in detail and also show some of the patients with DBS treatment during the pre‐lesion target evaluation period. An edited version of the recovered 1962 PD film is presented as a supplementary file accompanying this letter. A shorter clip from the same material has been published elsewhere.^7^

It seems that most stimulations were performed in or near the thalamus, and the films show excellent tremor suppression. But Sem‐Jacobsen also documents effects after DBS in other regions. The film presented here shows an example of stimulation close to the red nucleus (Fig. 1C). Sem‐Jacobsen reported general clinical improvement, also including rigidity in some patients,^2^, ^3^ and a published diagram showed a patient with striking improvement in bradykinesia as well as rigidity and tremor (5, see Fig. 1D). Tremor suppression may be obtained from several sites in this area, including different thalamic nuclei and the posterior subthalamic area with, eg, Zona incerta, prelemniscal radiations, and cerebellothalamic fibers. However, neither of these stimulation sites is effective against bradykinesia or rigidity. Stimulation of the red nucleus produces no such effect, but DBS targeted in the adjacent subthalamic nucleus usually produces a good general anti‐parkinsonian outcome.^8^ Several illustrations show that Sem‐Jacobsen tried stimulation at various sites, but the diagrams also document that some of the patients obtained good effect from both electrical stimulation and chemical lesion 10–15 mm lateral to the midline and below an imaginary line from the foramen of Monro to the pineal body.^2^, ^3^ This is the same region where electrodes are usually placed in modern subthalamic nucleus DBS surgery.^9^ With observed improvement in all parkinsonian motor features after stimulation close to the red nucleus, it is tempting to suggest that Sem‐Jacobsen in some of his patients tried temporary DBS in the subthalamic nucleus almost 40 years before this treatment was properly introduced.^8^

## Supporting information


**Video S1** Short clip from a film recorded by Dr. Carl Wilhelm Sem‐Jacobsen at Gaustad Psychiatric Hospital in Oslo, Norway, in the late 1950s or early 1960s. The first part shows glimpses from one of the first DBS operations performed on a patient with Parkinson's disease. The second part demonstrates tremor suppression over short periods in one patient as DBS is turned on (as red and green bulbs light up in the background panel) or off, and lasting tremor suppression by long‐term stimulation in another patient. Both patients were stimulated unilaterally, in the right hemisphere. Sem‐Jacobsen suggests that stimulation was performed close to the red nucleus. As discussed in the text, it is possible that the actual stimulation site was the subthalamic nucleus, which is located next to the red nucleus. The film was shown at a neurology meeting in 1962, has later gone missing, but was recently recovered. This and other films from the same material have now been donated to the National Medical Museum, which is part of the Norwegian Museum for Science and the Technologies in Oslo. A shorter clip from the same film material has been published elsewhere.^7^

## Data Availability

The data that support the findings of this study have been donated to and will be openly available from the National Medical Museum which is part of the Norwegian Museum for Science and the Technologies in Oslo, Norway.
